# Hemobilia-a rare complication after laparoscopic cholecystectomy-

**DOI:** 10.1186/s40792-020-00837-6

**Published:** 2020-05-05

**Authors:** Takehiro Abiko, Yuma Ebihara, Motoya Takeuchi, Hiroki Sakamoto, Hisato Homma, Satoshi Hirano

**Affiliations:** 1 Gastroenterological Surgery, Sapporo Kyoritsu Gorinbashi Hospital, 1-chome, Kawazoe 1-jo, Minami-ku, Sapporo, Hokkaido 0050802 Japan; 2 Department of Gastroenterological Surgery II, Hokkaido University Faculty of Medicine, North 15 West 7, Kita-ku, Sapporo, Hokkaido 0608638 Japan; 3 Gastroenterology, Sapporo Kyoritsu Gorinbashi Hospital, 1-chome, Kawazoe 1-jo, Minami-ku, Sapporo, Hokkaido 0050802 Japan

**Keywords:** Double cystic artery, Hemobilia, Laparoscopic cholecystectomy

## Abstract

**Background:**

Biliary bleeding is a condition reported by Sandblom as hemobilia. The most common cause of hemobilia is iatrogenicity. But it has also been reported as a rare complication after laparoscopic cholecystectomy (LC).

**Case presentation:**

A man in his 60s underwent a LC. He was taking a direct Xa inhibitor for paroxysmal atrial fibrillation (pAf) and had a history of thrombectomy. There was variation in the bifurcation of the hepatic artery and cystic artery. The right hepatic artery branches from the common hepatic artery by itself, and the cystic artery is double. He complained of right upper quadrant pain, nausea, and vomiting on the third postoperative day (3POD). Non-contrast computed tomography (CT) showed that a high absorption area was found to fill the common bile duct. Contrast CT showed no pseudoaneurysm formation. Ultimately, he was diagnosed with postoperative hemobilia. Angiographic examination selective for the cystic artery branching from the middle hepatic artery revealed leakage of the contrast agent and a micro-pseudoaneurysm.

**Conclusions:**

We encountered a case of hemobilia after LC. In this case, it was presumed that in addition to the chronic inflammatory changes of the gallbladder wall, extraordinary bifurcation of the hepatic artery and the cystic arteries and easy bleeding due to resumption of a direct Xa inhibitor synergistically caused a micro-pseudoaneurysm and postoperative hemobilia. It was difficult to identify the cause of hemobilia by contrast CT alone. Angiographic examination was useful for identifying and treating the causative artery and needs to perform aggressively.

## Background

Hemobilia, first reported by Sandblom [1], deals with bleeding into the biliary tract. (a) Severe right upper quadrant pain, (b) hematemesis and/or melena, and (c) elevated bilirubin are known as the three signs of hemobilia, as reported by Grove [2]. The most common cause of hemobilia is iatrogenicity [3]. Additionally, percutaneous treatment is often considered a cause for hemobilia, but it has also been reported as a rare complication after laparoscopic cholecystectomy (LC). Hemobilia is a major complication requiring angiographic examination and/or reoperation for treatment, so it needs to be widely known. Here, we report a case of hemobilia which required angiographic examination for diagnosis, caused by a micro-pseudoaneurysm of the cystic artery branching from the middle hepatic artery.

## Case presentation

**Fig. 1:**
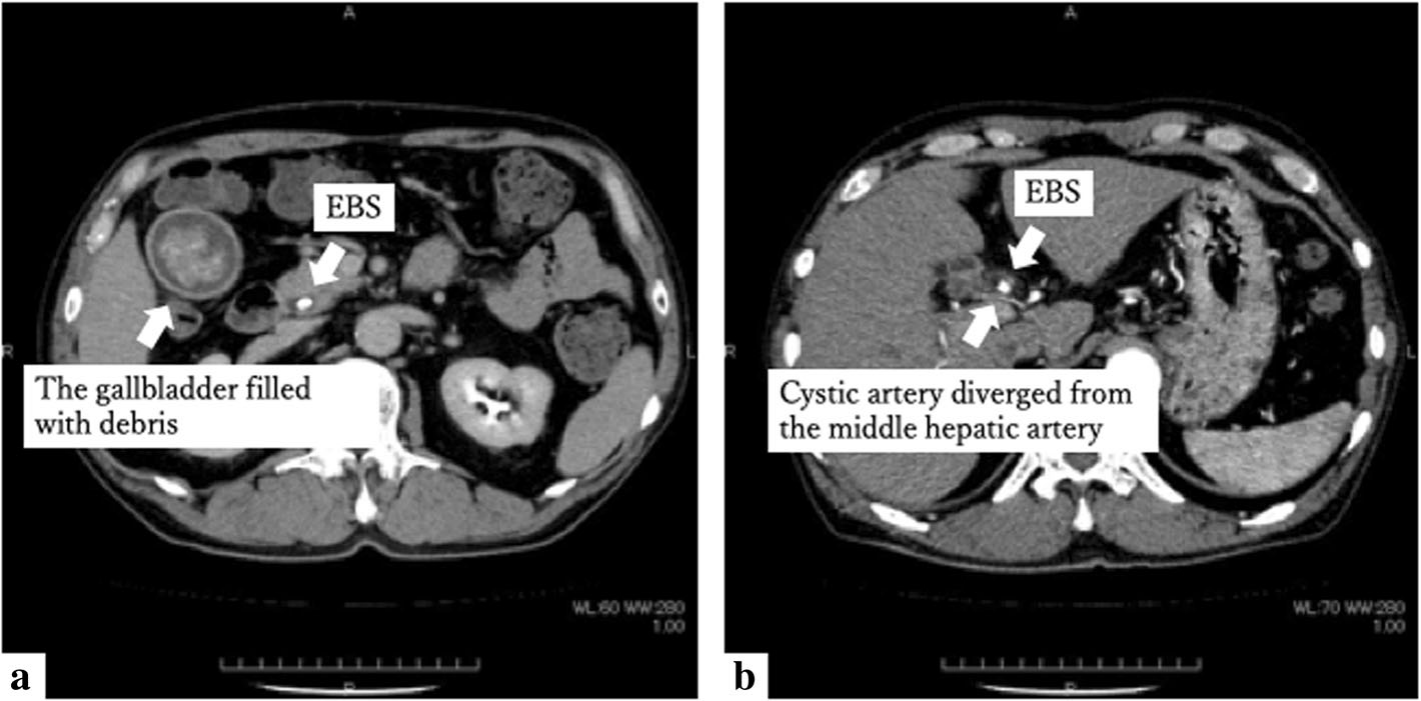
Contrast CT before LC. **a** The gallbladder was filled with debris. **b** Cystic artery diverged from the middle hepatic artery

A man in his 60s was admitted to our institution. He underwent LC for cholecystitis. He was taking a direct Xa inhibitor for paroxysmal atrial fibrillation (pAf) and had a history of thrombectomy for a right lower limb artery thrombotic occlusion. Contrast computed tomography (CT) revealed that the gallbladder wall was slightly thickened around the neck of the gallbladder. The gallbladder was filled with debris. Endoscopic biliary stenting (EBS) was inserted into the common bile duct (Fig. 1). EBS was removed 2 weeks before surgery, and endoscopic sphincterotomy (EST) was performed. No bile duct abnormalities were found by magnetic resonance cholangiopancreatography (MRCP).

The right hepatic artery branches from the common hepatic artery by itself, the middle hepatic artery branches from the left hepatic artery, and the cystic artery branches from the right hepatic artery. When considered in detail after surgery, another cystic artery had diverged from the middle hepatic artery (Fig. 1).

LC was performed at 3 ports. The gallbladder was tense highly inflamed at the neck. The gallbladder was peeled antegrade; the cystic artery branching from the right hepatic artery was identified and cut off after clipping. At this point, the cystic artery branching from the middle hepatic artery was not identified. Subtotal cholecystectomy was performed due to intense inflammation of the gallbladder neck. The operative time was 33 min, and the amount of blood loss was slight. Pathological results showed thickening of the wall, formation of Rokitansky-Aschoff sinus (RAS), and moderate infiltration of chronic inflammatory cells.

**Fig. 2:**
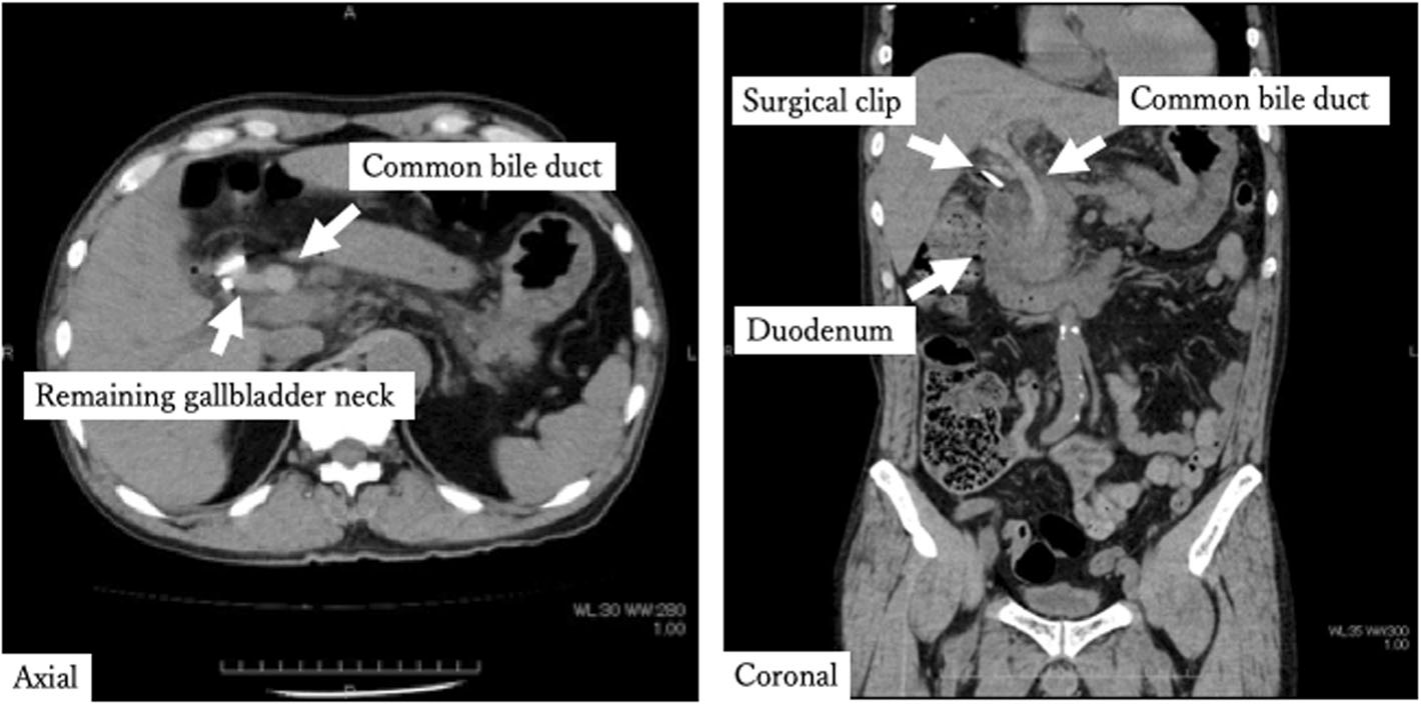
Non-contrast CT after LC. Non-contrast CT showed that a high absorption area filled the common bile duct

**Fig. 3:**
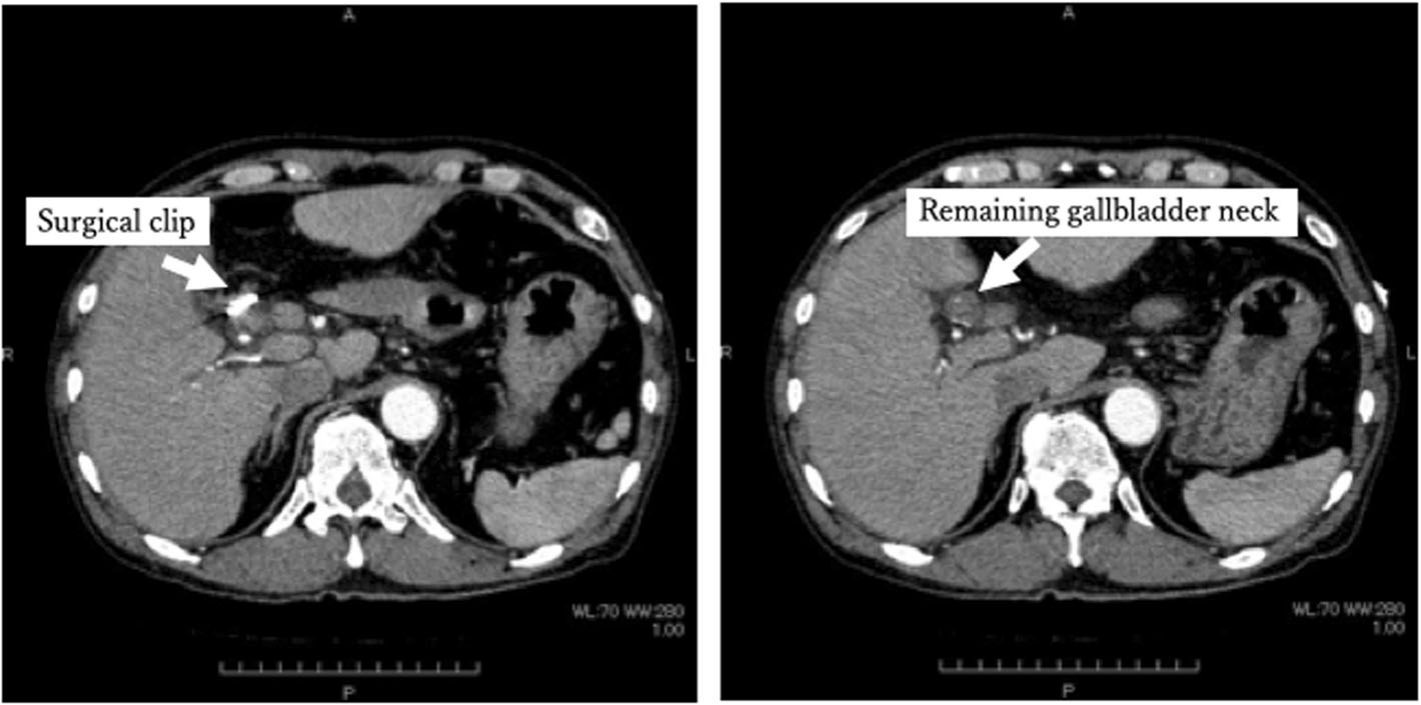
Contrast CT after LC. Contrast CT showed no leakage of the contrast agent and pseudoaneurysm formation

After confirming that there was no bleeding from the wound on the second postoperative day (2POD), the direct Xa inhibitor was resumed. The patient complained of right upper quadrant pain and vomiting on the third postoperative day (3POD). Blood sampling after onset shows an increase of hepatobiliary enzymes, but does not show progression of anemia (Table 1). Non-contrast CT was performed due to suspected residual stones and showed that a high absorption area filled the common bile duct (Fig. 2). Contrast CT showed no leakage of the contrast agent and pseudoaneurysm formation (Fig. 3). It was difficult to identify the cause of hemobilia by contrast CT alone. Postoperative hemobilia was considered to have formed a hematoma filling the common bile duct.

**Table 1:**
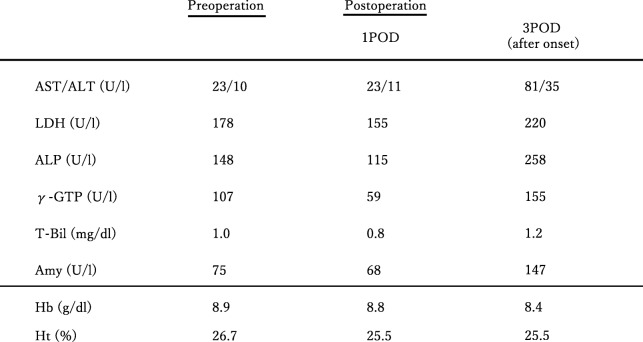
Laboratory data

It was deemed that multidisciplinary treatment concerning the insertion of an endoscopic nasobiliary drainage (ENBD) tube, angiographic examination, embolization, and reoperation for bleeding control was necessary, and the patient was transferred to an advanced medical institution.

**Fig. 4:**
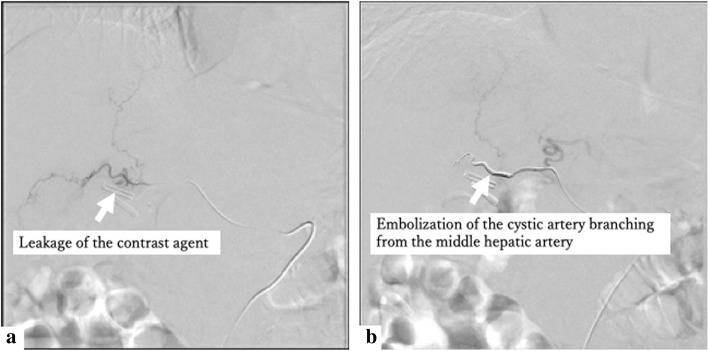
Angiographic examination. **a** Angiographic examination selective for the cystic artery branching from the middle hepatic artery revealed leakage of the contrast agent into the remaining lumen of the gallbladder neck and a micro-pseudoaneurysm. **b** Embolization of the cystic artery branching from the middle hepatic artery

Gastrointestinal endoscopy after transfer confirmed hematoma outflow from the Vater papilla. The patient was followed up with by having an ENBD tube inserted, but no subsequent bleeding was found. Angiography was performed before starting the direct Xa inhibitor. Angiographic examination selective for the cystic artery branching from the middle hepatic artery revealed leakage of the contrast agent and a micro-pseudoaneurysm, and embolization was performed (Fig. 4). The patient was discharged without rebleeding after resuming a direct Xa inhibitor.

## Discussion

Biliary bleeding is a condition reported by Sandblom as hemobilia [1]. Hemorrhage occurs in the bile duct, and blood flows from the Vater papilla into the gastrointestinal tract, causing hematemesis and melena. In addition, obstruction of the bile duct can cause obstructive jaundice, cholangitis, and pancreatitis due to impaired bile excretion. (a) Severe right upper quadrant pain, (b) hematemesis and/or melena, and (c) elevated bilirubin are known as the three signs of hemobilia, as reported by Grove [2]. Furthermore, Green et al. [3] reported that among 222 cases, the causes of hemobilia were iatrogenic (65%), inflammation (13%), tumor (7%), trauma (6%), and others (9%). To investigate the cause of hemobilia after LC, a PubMed search was conducted. After searching for keywords such as “Hemobilia,” “Postoperative,” and “Laparoscopic cholecystectomy,” 16 case reports (29 cases) were found [4–19] (Table 2). In these reports, the causes of hemobilia after LC was pseudoaneurysms of the hepatic artery or cystic artery stump. The most common treatment for hemobilia caused by pseudoaneurysms was embolization (23 cases), followed by operation (5 cases). In this case, diagnosis of hemobilia was possible by non-contrast and contrast CT, but identification of the cause artery was difficult. On the other hand, angiographic examination selective for the cystic artery branching from the middle hepatic artery revealed a micro-pseudoaneurysm, and embolization was performed. Angiographic examination is useful for identifying and treating the causative artery and needs to perform aggressively.

**Table 2:**
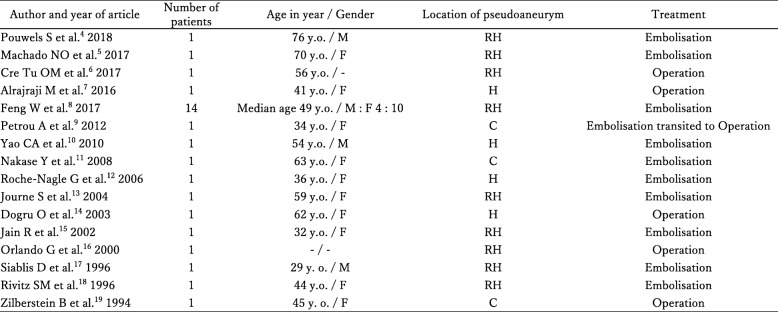
Case reports of hemobilia after laparoscopic cholecystectomy

*M* male, *F* female, *y.o* years old, *RH* right hepatic artery, *H* hepatic artery, *C* cystic artery

In examining the causes of micro-pseudoaneurysm formation and postoperative hemobilia, in this case, the following two points were considered: (a) the bifurcation of the hepatic artery and the cystic artery and (b) the timing of resumption of the direct Xa inhibitor.

Michels reported the bifurcation of the hepatic artery and the cystic artery. The hepatic artery’s bifurcation had been classified into 10 types [20]. In addition, there are variations in bifurcation of the cystic artery. Typically, the cystic artery is single. The double cystic arteries and the superficial and deep branches of the cystic artery which had separate origin are observed in 25% [20]. Andall presented the anatomical variations of cystic artery in literature review of over 9800 cases. That review has noted that in 8.9% (range 0.0–30.2 %) of cases, there were multiple cystic arteries present [21]. The cystic artery, in this case, had one branch each from the right hepatic artery and the middle hepatic artery. Moreover, in this operation, the cystic artery bifurcated from the right hepatic artery was identified and processed intraoperatively, but the cystic artery bifurcated from the middle hepatic artery was not recognized preoperatively, and intraoperative identification was not possible. As a result of the untreated cystic artery branching from the middle hepatic artery, it is presumed that a micro-pseudoaneurysm of the cystic artery was formed. It is necessary to examine CT image findings in detail and to perform surgery with anatomical variation in mind.

There is no unified guideline for when to resume direct Xa inhibitors after surgery. According to the package insert of the direct Xa inhibitor, in the case of venous thromboembolism in patients undergoing lower limb orthopedic surgery, the first administration should be performed 12 h after surgery and after confirming that there is no bleeding from surgical wounds. In this case, the timing of reinstatement was examined according to this description. Due to a history of right lower limb artery thrombotic occlusion, the postoperative withdrawal period of the direct Xa inhibitor was as short as possible. Since there was no bleeding from the wound, we concluded that there was no risk of post-bleeding. When the administration of the direct Xa inhibitor was resumed on the 2POD, hemobilia occurred on the 3POD. The postoperative course suggests that the resumption of oral administration of the direct Xa inhibitor was one of the causes of hemobilia.

## Conclusions

In conclusion, we experienced a case of hemobilia after LC. In this case, it was presumed that in addition to the mechanical stimulus of surgery added to the gallbladder wall with chronic inflammatory changes, extraordinary bifurcation of the hepatic and cystic arteries and easy bleeding due to resumption of a direct Xa inhibitor synergistically caused a micro-pseudoaneurysm and postoperative hemobilia. It was difficult to identify the cause of hemobilia by contrast CT alone. Angiographic examination was useful for identifying and treating the causative artery and needs to perform aggressively. Detailed confirmation of CT image findings and check of anatomical variation is important for surgeons.

## Data Availability

All datasets supporting the conclusions of this article are included within the article.

## Abbreviations

LC: Laparoscopic cholecystectomy
pAf: Paroxysmal atrial fibrillation
EBS: Endoscopic biliary stenting
EST: Endoscopic sphincterotomy
POD: Postoperative day
CT: Computed tomography
MRCP: Magnetic resonance cholangiopancreatography
RAS: Rokitansky-Aschoff sinus
ENBD: Endoscopic nasobiliary drainage
